# Pathophysiological Role of Primary Motor Cortex in Essential Tremor

**DOI:** 10.1002/mds.30197

**Published:** 2025-04-17

**Authors:** Daniele Birreci, Luca Angelini, Giulia Paparella, Davide Costa, Antonio Cannavacciuolo, Massimiliano Passaretti, Martina De Riggi, Simone Aloisio, Donato Colella, Andrea Guerra, Matteo Bologna

**Affiliations:** ^1^ Department of Human Neurosciences Sapienza University of Rome Rome Italy; ^2^ IRCCS Neuromed Pozzilli Italy; ^3^ Department of Clinical Neuroscience Karolinska Institutet Stockholm Sweden; ^4^ Parkinson and Movement Disorder Unit, Study Center on Neurodegeneration (CESNE) Department of Neuroscience University of Padua Padua Italy; ^5^ Padova Neuroscience Center University of Padua Padua Italy

**Keywords:** bradykinesia, essential tremor, motor control, primary motor cortex, transcranial magnetic stimulation

## Abstract

**Background:**

Essential tremor (ET) is one of the most prevalent movement disorders. However, the complete understanding of ET pathophysiology remains elusive.

**Objective:**

To explore the pathophysiological role of primary motor cortex (M1) in ET, specifically exploring its neurophysiological changes and their correlation with voluntary motor abnormalities.

**Methods:**

We recruited 30 ET patients and 18 healthy controls (HC). Evaluations were conducted on patients using clinical scales. Transcranial magnetic stimulation (TMS) was used to assess M1 excitability, including motor thresholds and motor evoked potentials (MEPs) input/output curve, together with intracortical excitability measures. Long‐term potentiation (LTP)‐like plasticity of M1 was tested using intermittent theta‐burst stimulation (iTBS). Objective assessments of tremor and voluntary movement execution during finger‐tapping were conducted through kinematic analysis. Finally, we explored the potential relationship between TMS, clinical, and kinematic data.

**Results:**

Compared with HC, ET patients had lower excitability, intracortical inhibition, and lower LTP‐like plasticity of M1. ET patients also exhibited slower finger‐tapping performance compared with HC. Among ET patients, the degree of movement slowing during finger‐tapping correlated with alterations in corticospinal excitability. Specifically, reduced M1 excitability was associated with lower finger‐tapping velocity. No other correlations were found.

**Conclusions:**

The study findings reveal neurophysiological alterations of M1 in ET and demonstrate correlations between excitability measures and voluntary motor performance. These results provide novel insight into the pathophysiology of ET, emphasizing the role of M1 changes in this condition. © 2025 The Author(s). *Movement Disorders* published by Wiley Periodicals LLC on behalf of International Parkinson and Movement Disorder Society.

Essential tremor (ET) is one of the most prevalent movement disorders. While the pivotal role of the cerebellum in ET pathophysiology is well‐recognized,^1^, ^2^, ^3^, ^4^, ^5^ only a limited number of experimental studies have employed transcranial magnetic stimulation (TMS) to investigate the contribution of the primary motor cortex (M1).^6^, ^7^, ^8^, ^9^, ^10^, ^11^, ^12^, ^13^ Specifically, these TMS studies explored excitability and plasticity mechanisms within M1 in ET, yielding variable results^6^, ^7^, ^8^, ^9^, ^10^, ^11^, ^12^, ^13^ (Table S1). Some studies reported increased corticospinal excitability^8^ and reduced M1 plasticity in ET,^7^, ^13^ while others observed normal corticospinal excitability and intracortical inhibitory mechanisms in this condition.^9^, ^10^ Hence, further studies are essential to investigate the neurophysiological changes of M1 in ET and to explore their potential relationship with clinical data. These investigations hold significant relevance, considering the central role of M1 as a primary recipient of cerebellar output.^14^

Patients with ET may exhibit subtle voluntary motor abnormalities, that is, movement slowness (bradykinesia),^15^ within the spectrum of so‐called “soft signs”.^16^, ^17^, ^18^, ^19^, ^20^, ^21^, ^22^, ^23^ A previous study has shown that slowed voluntary movement execution in ET is linked to disrupted functional connectivity within cerebellar circuits, basal ganglia, and sensorimotor areas.^24^ A potential link has also been observed between subtle changes in central dopaminergic tone and altered voluntary movement execution in ET, similar to what occurs in Parkinson's disease (PD).^25^, ^26^ Given the central role of M1 in motor control, we aimed to investigate whether changes in M1, such as alterations in excitability and plasticity, contribute to the voluntary movement abnormalities observed in ET patients, as previously demonstrated in PD.^27^, ^28^

Besides subtle voluntary movements abnormalities, ET patients may also exhibit various soft signs, such as rest tremor and others.^18^, ^20^, ^29^ In this study, we also aimed to explore the neurophysiological parameters in relation to the considerable heterogeneity of ET and the presence of soft signs.^18^, ^20^, ^29^ In addition to slowed voluntary movement, we focused on rest tremor, as both are common parkinsonian signs in ET, although their potential relationship in ET remains largely unknown.

In this study, we first performed a comprehensive clinical evaluation of ET patients, assessing key M1 excitability and plasticity parameters using single‐ and paired‐pulse TMS protocols alongside intermittent theta‐burst stimulation (iTBS). Tremor and repetitive finger‐tapping were objectively analyzed using an optoelectronic system. Data from ET patients were compared with those from healthy subjects. Finally, we examined potential relationships between clinical findings, TMS parameters, and kinematic measures in patients.

## 1 Methods

### 1.1 Participants and Clinical Assessment

Thirty patients diagnosed with ET and 18 healthy controls (HC), all right‐handed, were consecutively enrolled in the study (Table 1). The patient diagnosis was based on consensus criteria.^22^ Among them, 7 (23%) were classified as pure ET, while 23 (77%) were categorized as ET‐plus (Table 1). As part of the clinical evaluation, all patients underwent brain magnetic resonance imaging (MRI) and laboratory screening to rule out other specific etiologies of tremor.^30^ Individuals taking medications that could potentially impact the central nervous system discontinued their therapy at least 72 h before the evaluation.^11^, ^31^, ^32^ The Fahn–Tolosa–Marin Tremor Rating Scale (FTM‐TRS)^33^ and the motor section (Part III) of the Movement Disorder Society‐sponsored revision of the Unified Parkinson's Disease Rating Scale (MDS‐UPDRS)^34^, ^35^ were administered to the patients. Global cognition was assessed in the whole sample with the Montreal Cognitive Assessment (MoCA)^36^ and the Frontal Assessment Battery (FAB).^37^ All experimental procedures were conducted in accordance with the Declaration of Helsinki and international safety guidelines^38^ and approved by the local ethics committee. All participants gave informed consent prior to participating in the study.

**TABLE 1:** Demographic and clinical data of healthy controls (HC), essential tremor patients (ET), and the two ET subgroups: ET‐slowness and ET no‐slowness

Parameter	HC [n = 18]	ET [n = 30]	*P*	ET‐s [n = 15]	ET‐ns [n = 15]	*P*
Age (years)	68.11 ± 8.85	66.56 ± 10.57	0.99	66.27 ± 8.97	66.97 ± 12.28	0.53
Sex	7 M (38.9%)	20 M (66.7%)	0.08	9 M (60.0%)	11 M (73.3%)	0.70
Tremor duration (years)	–	17.77 ± 12.71	–	20.20 ± 14.72	15.33 ± 10.25	0.66
FTM‐TRS	–	24.87 ± 14.27	–	29.87 ± 17.13	19.87 ± 8.66	0.11
MDS‐UPDRS‐III	–	9.57 ± 6.98	–	11.47 ± 7.90	7.67 ± 5.54	0.18
MoCA	25.50 ± 3.17	24.43 ± 2.89	0.24	23.47 ± 2.97	25.40 ± 2.53	0.04
FAB	16.44 ± 2.12	16.63 ± 1.75	0.96	16.33 ± 2.06	16.93 ± 1.39	0.34
Soft signs						
RT	–	13 (43.3%)	–	9 (60.0%)	4 (26.7%)	0.14
MCI	–	11 (36.7%)	–	8 (53.3%)	3 (20.0%)	0.13
QD	–	12 (40.0%)	–	6 (40.0%)	6 (40.0%)	1.00
ITG	–	5 (16.7%)	–	4 (26.7%)	1 (6.7%)	0.33
Therapy						
Propranolol	–	14 (46.7%)	–	8 (53.3%)	6 (40.0%)	0.72
Topiramate	–	3 (10.0%)	–	2 (13.3%)	1 (6.7%)	1.00
BDZ	–	5 (16.7%)	–	3 (20.0%)	2 (13.3%)	1.00
Gabapentin	–	5 (16.7%)	–	3 (20.0%)	2 (13.3%)	1.00
No therapy	–	9 (30.0%)	–	4 (26.7%)	5 (33.3%)	1.00

*Note*: Values are expressed as mean ± standard deviation (SD). The number of participants in each subgroup are in parentheses in the column headers, and the percentages are stated within the table rows. For each soft sign and drug, the number of patients is specified. *P*‐values were calculated using the Mann–Whitney U test or the Fisher's exact test where appropriate. After false discovery rate correction, none of the values reached statistical significance.

Abbreviations: BDZ, benzodiazepines; ET, essential tremor; ET‐ns, essential tremor no‐slowness; ET‐s, essential tremor‐slowness; FAB, Frontal Assessment Battery; FTM‐TRS, Fahn–Tolosa–Marin Tremor Rating Scale; HC, healthy controls; ITG, impaired tandem gait; M, male; MCI, mild cognitive impairment; MDS‐UPDRS‐III, Movement Disorder Society‐sponsored revision of the Unified Parkinson's Disease Rating Scale (Part III); MoCA, Montreal Cognitive Assessment; QD, questionable dystonia; RT, rest tremor.

### 1.2 TMS

Single‐ and paired‐pulse TMS was administered using a Magstim BiStim2 with eight‐shaped coil delivering monophasic pulses (Magstim Co. Ltd, Whitland, UK). The coil was positioned tangentially to the scalp, with the handle directed posteriorly and laterally at a 45° angle from the midline.^39^ The hotspot of the dominant hand's first dorsal interosseous (FDI) muscle was initially identified on the contralateral M1. Resting motor threshold (RMT), active motor threshold (AMT), and the stimulation intensity required to elicit motor evoked potentials (MEPs) with an amplitude of approximately 1 mV (MT1mV) were then determined.^39^ The input/output (I/O) curve was assessed by administering 10 single TMS pulses at five distinct stimulation intensities, ranging from 100% to 180% RMT with increments of 20%.^39^ The sequence of the tested intensities was randomized.^40^ The I/O slope for MEPs was calculated using a linear regression analysis between stimulus intensity and MEP amplitude.^41^, ^42^

Short‐interval intracortical inhibition (SICI) and short‐latency afferent inhibition (SAI) were assessed using standardized protocols.^39^, ^43^ SICI was tested by administering paired TMS pulses with a subthreshold conditioning stimulus at 80% AMT, a suprathreshold test stimulus at MT1mV, and interstimulus intervals (ISI) of 2 and 4 ms. For SAI, stimulation of the median nerve at the wrist was conducted using rectangular electrical pulses (Digitimer DS7 model; Digitimer, Welwyn Garden City, UK) at the intensity inducing a painless contraction of the thumb and followed by a single TMS pulse at MT1mV with ISI of 22 and 24 ms. Fifteen conditioned MEPs were recorded for SICI (2 and 4 ms) and SAI (22 and 24 ms), which were randomized with 15 unconditioned MEPs elicited at MT1mV intensity.^39^, ^43^

The long‐term potentiation (LTP)‐like plasticity of M1 was investigated using iTBS.^44^ The iTBS protocol was administered using a biphasic stimulator (Magstim SuperRapid; Magstim Co. Ltd) connected to an eight‐shaped coil over the FDI hotspot, as described in previous studies.^41^, ^42^ The stimulation intensity was set at 80% AMT. Fifteen MEPs evoked by single TMS pulses at MT1mV were recorded before (T0) and 5 (T1), 15 (T2), and 30 (T3) min after iTBS. Electromyography (EMG) signals were amplified and filtered (20 Hz–1 kHz) using a Digitimer D360 (Digitimer), stored on a laboratory computer (sampling frequency of 5 kHz) via an AD1401 plus analog‐to‐digital converter (Cambridge Electronic Design, Milton, UK), and analyzed using dedicated software (Signal Version 5.08, Cambridge Electronic Design). Then, peak‐to‐peak MEP amplitudes were averaged for each condition. SICI and SAI were expressed as the ratio of conditioned to unconditioned MEP amplitudes.

### 1.3 Kinematic Analysis

The assessment was conducted using an optoelectronic system as described previously.^17^, ^19^, ^21^, ^26^ In ET patients, upper limb postural tremor was recorded with the arms outstretched in front of the chest (posture 1‐P1) and with the arms flexed at the elbows, that is, lateral “wing beating” posture (posture 2‐P2), with three 45‐s recordings for each position. Rest tremor was assessed with patients seated comfortably on a chair, arms fully relaxed and placed on a table in front of them, following the same recording protocol. The kinetic tremor was recorded for three 15‐s sessions with patients performing a pointing task, as previously described.^45^ The root mean square (RMS) of the acceleration traces of the reference markers in three‐dimensional space was measured to determine the magnitude of tremor and expressed in GRMS^2. Power spectra were calculated through fast Fourier transform. The dominant frequency peak (Hz) of tremor was measured, and the tremor amplitude was then determined by measuring the tremor power at the individual frequency peak ± 1 Hz.^20^ The amplitude and frequency of postural tremor were calculated as the mean of the P1 and P2 measurements. For kinetic tremor (KT), we used an algorithm that determined deceleration/acceleration ratio (D/A) (i.e., duration of the deceleration phase/duration of the acceleration phase) and curvature index (CI) (i.e., arm endpoint average path length/length of a straight line joining the initial and final positions). The D/A and CI are considered indices of movement homogeneity, and reflect intention tremor.^19^, ^20^, ^21^, ^45^ Since no substantial side differences were found for the tremor parameters, the average of values between the two sides was considered for the analysis (Tables 2 and S2).

**TABLE 2:** Kinematic and transcranial magnetic stimulation measures in healthy controls (HC), essential tremor patients (ET), and the two ET subgroups: ET‐slowness and ET no‐slowness

Parameter	HC [n = 18]	ET [n = 30]	*P*	ET‐s [n = 15]	ET‐ns [n = 15]	*P*
RMT	45.65 ± 5.14	51.29 ± 8.39	**0.01**	55.38 ± 7.31	47.20 ± 7.51	**0.01**
AMT	37.32 ± 3.52	41.67 ± 7.38	0.04	45.40 ± 6.71	37.93 ± 6.16	**<0.01**
I/O slope	3.65 ± 2.21	3.24 ± 2.18	0.46	2.42 ± 1.49	4.06 ± 2.49	**0.02**
SICI						
2 ms	0.47 ± 0.19	0.65 ± 0.33	0.25	0.58 ± 0.25	0.73 ± 0.38	1.00
4 ms	0.59 ± 0.23	0.79 ± 0.36	0.21	0.76 ± 0.39	0.83 ± 0.35	1.00
SAI						
22 ms	0.60 ± 0.47	0.51 ± 0.22	1.00	0.49 ± 0.23	0.53 ± 0.21	0.97
24 ms	0.71 ± 0.44	0.56 ± 0.26	0.86	0.56 ± 0.26	0.56 ± 0.26	1.00
Post iTBS						
5 min	1.20 ± 0.23	0.96 ± 0.34	0.18	0.97 ± 0.35	0.96 ± 0.34	1.00
15 min	1.46 ± 0.45	1.03 ± 0.43	0.03	0.94 ± 0.33	1.12 ± 0.51	0.84
30 min	1.33 ± 0.32	1.08 ± 0.48	0.86	1.18 ± 0.47	0.98 ± 0.48	0.87
Rest tremor						
Amplitude	–	0.06 ± 0.06	–	0.07 ± 0.06	0.04 ± 0.04	0.43
Frequency	–	6.10 ± 0.97	–	6.16 ± 1.10	5.98 ± 0.74	0.94
Postural tremor						
Amplitude	–	0.10 ± 0.07	–	0.11 ± 0.07	0.09 ± 0.7	0.47
Frequency	‐	5.96 ± 1.23	–	5.79 ± 1.35	6.15 ± 1.11	0.24
Kinetic tremor						
CI	–	1.08 ± 0.07	–	1.09 ± 0.08	1.06 ± 0.05	0.68
D/A	–	0.53 ± 0.16	–	0.55 ± 0.19	0.51 ± 0.13	0.63
Finger‐tapping						
Movements (n)	48.22 ± 13.31	41.61 ± 11.03	0.12	41.14 ± 11.35	42.08 ± 11.07	1.00
Velocity	1189.07 ± 149.28	996.26 ± 219.76	**<0.01**	825.68 ± 179.73	1166.85 ± 73.33	**<0.01**
Amplitude	45.48 ± 11.26	47.16 ± 10.61	0.55	44.34 ± 10.85	49.98 ± 9.92	0.16
CV	0.11 ± 0.05	0.11 ± 0.05	0.72	0.11 ± 0.04	0.10 ± 0.05	0.20
Velocity slope	−4.10 ± 4.85	−4.17 ± 5.61	0.89	−3.56 ± 4.75	−4.78 ± 6.46	0.37
Amplitude slope	−0.11 ± 0.18	−0.13 ± 0.30	0.91	−0.09 ± 0.24	−0.18 ± 0.35	0.60

*Note*: Values are expressed as mean ± standard deviation (SD). The number of participants in each subgroup are in parentheses in the column headers. Short‐interval intracortical inhibition (SICI), short‐latency afferent inhibition (SAI), and post‐intermittent theta‐burst stimulation (iTBS) values are expressed as the ratio of conditioned to unconditioned motor evoked potential amplitudes. Tremor values are expressed as the mean of both sides, and finger‐tapping values refer to the right (dominant) hand. *P*‐values for SICI, SAI, and synaptic plasticity were obtained from post‐hoc tests performed with Bonferroni correction, while the other *P*‐values were calculated using the Mann–Whitney U test. Only values that are significant after false discovery rate correction are highlighted in bold.

Abbreviations: AMT, active motor threshold; CI, curvature index; CV, coefficient of variation; D/A, deceleration/acceleration ratio; ET, essential tremor; ET‐ns, essential tremor no‐slowness; ET‐s, essential tremor‐slowness; HC, healthy controls; I/O slope, input/output slope of motor evoked potentials; iTBS, intermittent theta‐burst stimulation; RMT, resting motor threshold; SAI, short‐latency afferent inhibition; SICI, short‐interval intracortical inhibition.

Participants were also instructed to execute finger‐tapping.^17^, ^19^, ^21^, ^26^ Three blocks of movements, each lasting 15 s, were recorded. To quantify finger‐tapping, linear regression techniques were employed to ascertain the movement amplitude, velocity, and the decrease in amplitude and velocity during the movement repetition (i.e., sequence effect).^20^, ^21^ Additionally, the movement rhythm was measured by the coefficient of variation (CV) of the inter‐taps intervals^20^, ^21^ (Table 2). Regarding finger‐tapping kinematics, values obtained from the right (dominant) side, which corresponded to the left M1 tested with TMS, were considered for the analysis (Tables 2 and S2). Tremor and motion analysis were conducted using a dedicated software (SMART Analyzer; BTS Bioengineering, Milan, Italy).

### 1.4 Statistical Analysis

The Mann–Whitney U test was used to assess potential differences in clinical‐demographic data, as well as TMS and kinematic parameters between groups. Sex and other qualitative variables expressed as percentages were compared using Fisher's exact test. A repeated measures analysis of variance (rmANOVA) with the factors “GROUP” (ET and HC) and “STIMULUS INTENSITY” (100%, 120%, 140%, 160%, 180% RMT) was used to evaluate potential differences in the I/O curves. Similarly, rmANOVAs were employed to compare SICI and SAI, with the factors “GROUP” (ET and HC) and “ISI” (2 and 4 ms for SICI, 22 and 24 ms for SAI), and to assess the effects of iTBS, with the factors “GROUP” (ET and HC) and “MEASUREMENT TIME” (T1, T2, and T3). For the latter, MEP amplitude recorded at T1, T2, and T3 was normalized to the T0 value by calculating the ratio of each measurement to T0. Post‐hoc analysis in the various rmANOVAs was performed using Bonferroni's test. Greenhouse–Geisser corrections were applied whenever the Mauchly test identified violations of sphericity. Spearman's correlation coefficient was used to examine the potential relationship between clinical data and neurophysiological measurements (kinematic and TMS parameters). A median split procedure was also applied to classify ET‐slowness (ET‐s) and ET no‐slowness (ET‐ns) subgroups, and a between‐group analysis was conducted.^46^ Additionally, a separate between‐group analysis was conducted by dividing patients into ET‐rest tremor (ET‐r) and ET no‐rest tremor (ET‐nr) subgroups. Results are presented as mean values ±1 standard deviation (SD) unless otherwise stated. The significance level was set at *P* < 0.05. False discovery rate (FDR) correction for multiple comparisons was applied.^47^ Data were analyzed using IBM SPSS Statistics for Windows, Version 26 (IBM Corp., Armonk, NY, USA).

## 2 Results

### 2.1 ET versus HC

#### 2.1.1 Demographic and Clinical Characteristics

No significant differences were observed between ET patients and HC in terms of age, sex, MoCA, and FAB scores (Table 1).

#### 2.1.2 TMS: Motor Thresholds and I/O Curve

**FIG. 1:**
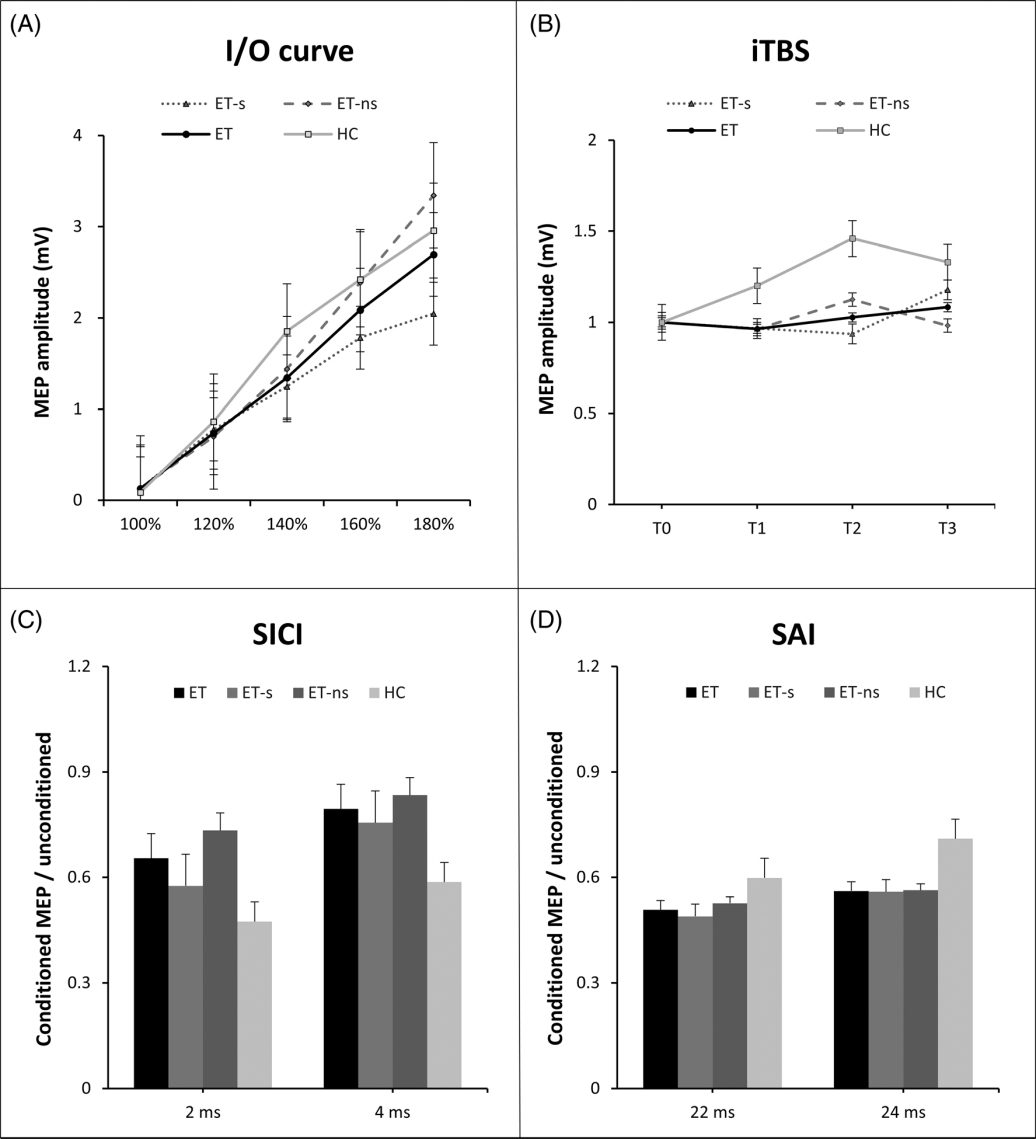
Neurophysiological measurements obtained by transcranial magnetic stimulation (TMS) techniques in healthy controls (HC), essential tremor patients (ET), and the two ET subgroups: ET‐slowness (ET‐s) and ET no‐slowness (ET‐ns). (A) Input/output (I/O) curve of motor evoked potentials (MEPs). The y‐axis represents the amplitude of MEPs expressed in mV. The x‐axis represents the five stimulation intensities tested (100%, 120%, 140%, 160%, and 180% of resting motor threshold). (B) Changes in MEP amplitude after intermittent theta‐burst stimulation (iTBS). The y‐axis represents the amplitude of MEPs normalized to the baseline amplitude (before iTBS, T0). The x‐axis represents the values collected at three different measurement times: 5 (T1), 15 (T2), and 30 min (T3) after iTBS. (C, D) Short‐interval intracortical inhibition (SICI) and short‐latency afferent inhibition (SAI) values. The y‐axes represent the ratio between conditioned and unconditioned MEP amplitudes, while the x‐axes represent the inter‐stimulus intervals (ISI) tested (2 and 4 ms for SICI, and 22 and 24 ms for SAI) in different subgroups. Vertical bars indicate the standard error of the mean.

ET patients showed higher RMT, indicating reduced M1 excitability, compared with HC (Table 2). The I/O curve did not differ between ET and HC, as indicated by no significance of factor “GROUP” [*F*(1, 46) = 0.86, *P* = 0.36] and of interaction “GROUP × STIMULUS INTENSITY” [*F*(4,184) = 0.82, *P* = 0.51]. As expected, the factor “STIMULUS INTENSITY” was significant [*F*(4,184) = 88.35, *P* < 0.001] (Fig. 1).

#### 2.1.3 TMS: SICI And SAI

The rmANOVA of SICI revealed a significant effect of the factors “GROUP” [*F*(1, 46) = 5.78, *P* = 0.02] and “ISI” [*F*(1, 46) = 9.10, *P* < 0.01], with reduced SICI in ET patients compared with HC, and higher SICI at ISI 2 ms than at ISI 4 ms. However, the “GROUP × ISI” interaction for SICI was not significant [*F*(1,46) = 0.12, *P* = 0.73] (Fig. 1, Table 2). The rmANOVA of SAI revealed, as expected, a significant effect of the “ISI” [*F*(1,46) = 8.4, *P* < 0.01], with higher SAI at ISI 22 ms than at ISI 24 ms. No significance was observed for the “GROUP” [*F*(1,46) = 1.58, *P* = 0.22] and the interaction “GROUP × ISI” [*F*(1,46) = 1.08, *P* = 0.30] (Fig. 1, Table 2).

#### 2.1.4 TMS: iTBS After‐Effects

The rmANOVA of the effect of iTBS on MEPs amplitude revealed a significance of the factor “GROUP” [*F*(1,45) = 10.7, *P* < 0.01], with reduced iTBS effect in ET patients compared with HC. The factor “MEASUREMENT TIME” was also significant [*F*(2,90) = 3.70, *P* = 0.03], as expected, with a trend of greater MEP facilitation post‐iTBS at T2 than T1, which did not survive FDR correction. No significant “GROUP × MEASUREMENT TIME” interaction emerged [*F*(2,90) = 1.57, *P* = 0.21] (Fig. 1, Table 2).

#### 2.1.5 Movement Kinematics

Kinematic analysis in ET patients revealed a mean postural tremor amplitude of 0.10 ± 0.07 GRMS^2 and postural tremor frequency of 5.96 ± 1.23 Hz, rest tremor amplitude of 0.06 ± 0.06 GRMS^2 and rest tremor frequency of 6.10 ± 0.97 Hz, CI of 1.08 ± 0.07, and D/A of 0.53 ± 0.16. ET patients exhibited slower movement velocity compared with HC (*P* < 0.01), with no differences in the other movement parameters (Tables 2 and S2).

#### 2.1.6 Correlation Analysis

**FIG. 2:**
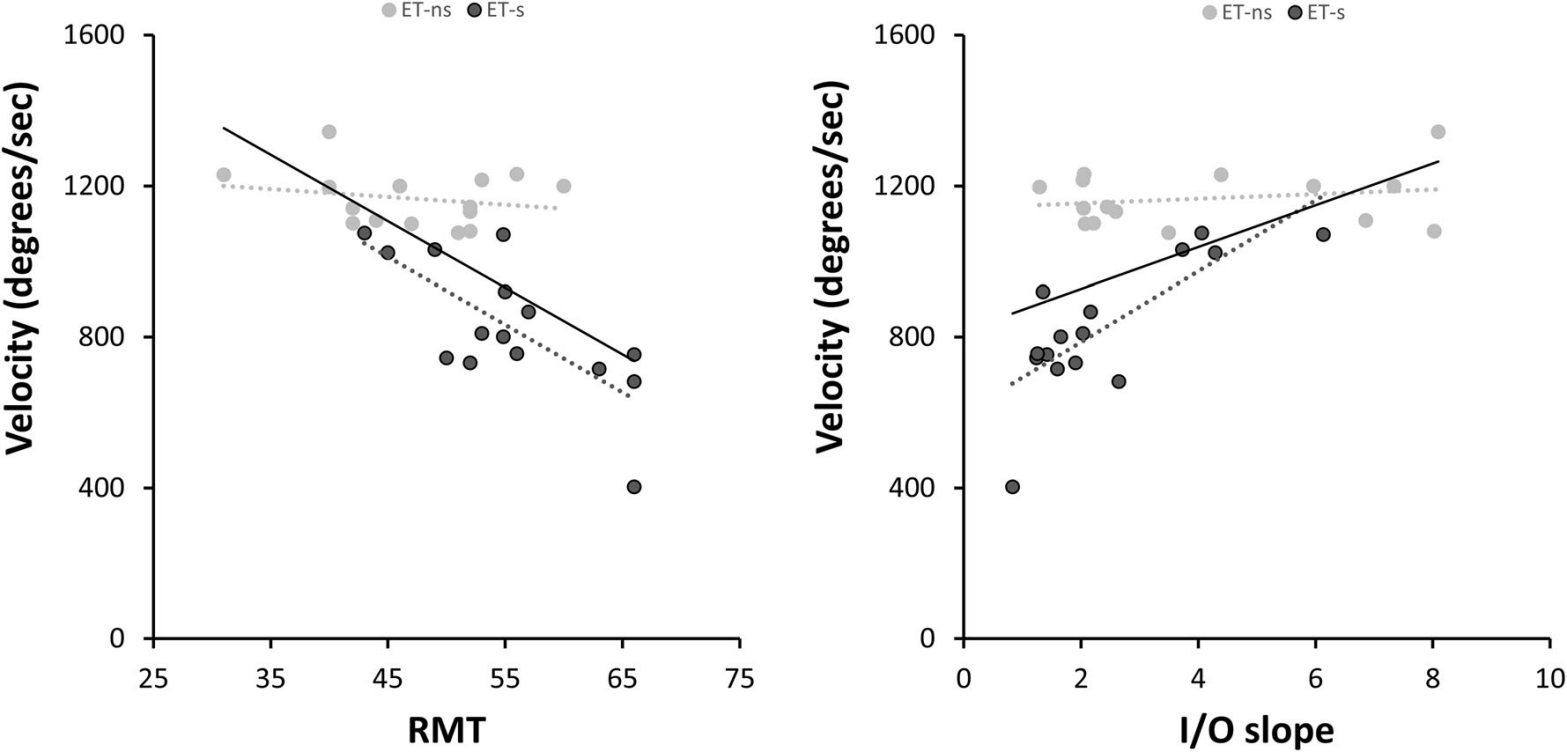
Correlations between kinematic values and neurophysiological parameters in essential tremor (ET) subgroups. The solid lines represent the trend lines for the entire ET group; the dark grey dotted lines represent the trend line for essential tremor‐slowness (ET‐s) patients; and the light grey dotted lines represent the trend line for essential tremor no‐slowness (ET‐ns) patients. RMT, resting motor threshold; I/O slope, input/output slope of motor evoked potentials.

In ET patients, movement velocity negatively correlated with rest motor threshold (RMT: rho = −0.54, *P* < 0.01) and positively correlated with the I/O slope (*r* = 0.51, *P* < 0.01), indicating that lower velocities corresponded to higher motor thresholds and flatter I/O curves, that is, lower cortical excitability (Fig. 2). No correlations were found between other TMS measurements and kinematic variables.

### 2.2 ET‐s versus ET‐ns

**FIG. 3:**
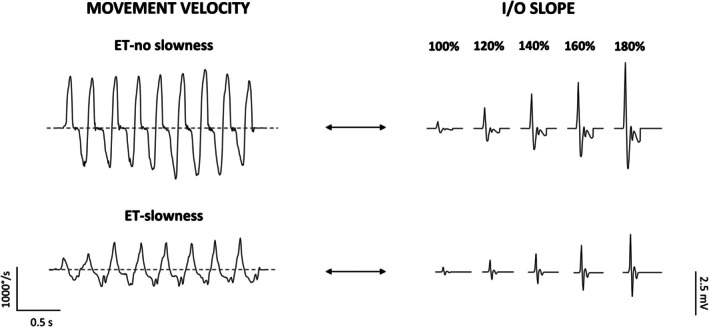
Schematic representation of the relationship between movement velocity and the slope of the input/output (I/O) curve. The left side of the image illustrates the movement velocity during the first finger‐tapping movements performed by the patients, while the right side depicts the I/O recruitment curve of motor evoked potentials at 100%, 120%, 140%, 160%, and 180% of the resting motor threshold. Notably, essential tremor (ET) patients without movement slowness exhibit a steeper I/O curve, whereas ET patients with movement slowness demonstrate a flatter I/O curve.

No differences between groups were demonstrated regarding age, sex, and other clinical scores (all *P* > 0.05), except for a trend towards lower MoCA scores in ET‐s (Table 1). Higher RMT and AMT were observed in ET‐s, indicating reduced cortical excitability, compared with ET‐ns (Table 2). In addition, when analyzing the I/O curves, significant effect was observed for the “STIMULUS INTENSITY” factor [*F*(4,112) = 58.50, *P* < 0.01] and for the “STIMULUS INTENSITY × GROUP” interaction [*F*(4,112) = 4.44, *P* < 0.01]. However, no effect was observed for the “GROUP” factor (Fig. 1). Regarding SICI, SAI, and iTBS effects, no differences were observed between ET‐s and ET‐ns (all *P* > 0.05, Table 2 and S3). None of the kinematic tremor or movement parameters, except for velocity, differed between the two groups (Table 2). Finally, significant correlations emerged between movement velocity and corticospinal excitability measures in the ET‐s subgroup (RMT: rho = −0.65, *P* < 0.01, AMT: rho = −0.70, *P* < 0.01, I/O slope: rho = 0.66, *P* = 0.01), with lower velocity associated with lower corticospinal excitability (Figs. 2 and 3). No other correlations were found between the remainder of the clinical variables, including MoCA and FAB scores, movement kinematics, and TMS parameters (all *P* > 0.05).

### 2.3 ET‐r versus ET‐nr

Thirteen patients (43%) had rest tremor, while 17 (57%) did not. No significant differences were observed between ET‐r and ET‐nr regarding age, sex, disease duration, or other clinical scores (all *P* > 0.05). Patients with ET‐r showed greater postural tremor amplitude compared with ET‐nr (*P* = 0.04), but this difference did not survive FDR correction. No other kinematic parameters, including tremor and finger‐tapping measures, differed between groups. Regarding neurophysiological measures, no significant differences were found in RMT, AMT, I/O curve, SICI, SAI, or iTBS‐induced plasticity effects between ET‐r and ET‐nr (all *P* > 0.05) (Table S4). Finally, no correlation was found between rest tremor amplitude and any of the neurophysiological parameters analyzed.

## 3 Discussion

We herein performed a detailed neurophysiological investigation of M1 in ET. Compared with HC, the major findings in ET were: (1) lower M1 excitability, (2) reduced intracortical inhibition, as assessed by SICI, and (3) reduced LTP‐like plasticity of M1, as assessed by iTBS. Our results confirmed that ET patients exhibited reduced movement velocity during finger‐tapping.^16^, ^19^, ^20^, ^26^ The correlation and subgroup analyses demonstrated a relationship between decreased M1 excitability and slowed movement execution in ET patients.

### 3.1 Neurophysiological Changes of M1 in ET

Concerning lower M1 excitability, that is, higher RMT values, in ET patients compared with HC, previous studies provided conflicting results^6^, ^7^, ^8^, ^9^, ^10^, ^12^, ^13^ (Table S1). Some studies indicate normal results,^9^, ^10^ while others report increased corticospinal excitability in ET.^8^ These discrepancies may be due to the relatively small sample size in prior studies,^7^, ^9^, ^12^, ^13^ methodological factors, such as the use of a rounded coil,^9^ which is known to be less focal compared with an eight‐shaped coil,^39^ or alternatively the choice to target the abductor digiti minimi muscle,^8^ which has a less reproducible cortical hotspot compared with the FDI.^39^, ^48^, ^49^ Despite changes in motor thresholds, there was no difference in the I/O curve between groups, possibly indicating the different neurophysiological basis of these measures. RMT primarily reflects the excitability of cortico‐cortical neurons, while the I/O curve more specifically represents the excitability of corticospinal fibers, largely influenced by glutamatergic drive.^50^, ^51^ In this regard, an MRI spectroscopy study has revealed a positive correlation between the slope of the I/O curve and cortical glutamate levels in the motor cortex.^52^ Our finding of normal I/O curve in ET agrees with the evidence suggesting normal glutamate levels in this condition.^53^, ^54^

We also found reduced intracortical inhibition (lower SICI) in ET compared with HC. SICI is a well‐known neurophysiological measure of GABA‐Aergic neurotransmission within M1.^31^ Despite previous studies examining this parameter not identifying differences between ET and HC,^7^, ^9^ our result may align with the hypothesis of GABAergic dysfunction in ET,^55^, ^56^ as observed within the deep cerebellar nuclei.^57^, ^58^, ^59^, ^60^ Notably, a study employing positron emission tomography (PET) with [^11^C]‐flumazenil demonstrated less availability of GABAergic receptors not only in the cerebellum but also in the ventrolateral thalamus and lateral premotor cortex in ET patients.^61^ Furthermore, this GABA defect seems to progress over the disease course.^57^, ^59^ The presence of GABAergic dysfunction across both subcortical and cortical regions suggests that similar mechanisms may occur at the level of M1. Regarding SAI, in this study we found similar values between ET and HC, suggesting that cholinergic circuits in M1 are intact in ET. Our SAI values in ET patients are comparable with those that emerged in a recent study, addressing drug effects on M1 neurophysiological measurements.^11^

In our study we also tested M1 plasticity changes in ET. We specifically tested iTBS after‐effects and observed a reduced MEP facilitation in ET compared with HC. iTBS‐induced effects are known to depend on both GABAergic activity and calcium dynamics within M1.^44^, ^62^ Similarly, reduced cortical plasticity in ET appears to be primarily associated with GABAergic circuits and calcium dynamics dysfunction.^63^, ^64^, ^65^ Conversely, microstructural alterations of the corticospinal tract do not appear to influence synaptic plasticity.^66^ Previous studies demonstrated impaired M1 plasticity in ET, showing reduced effects of continuous TBS (cTBS)^7^, ^13^ and no M1 excitability potentiation following paired associative stimulation (PAS) in ET patients^66^. However, one study reported no differences in PAS‐induced synaptic plasticity between HC and ET patients.^67^ Our findings, together with previous studies, support the hypothesis of impaired motor plasticity in ET, also aligning with evidence of disrupted motor learning in ET patients.^68^, ^69^

### 3.2 Neurophysiological Correlates of Voluntary Movement Execution in ET

**FIG. 4:**
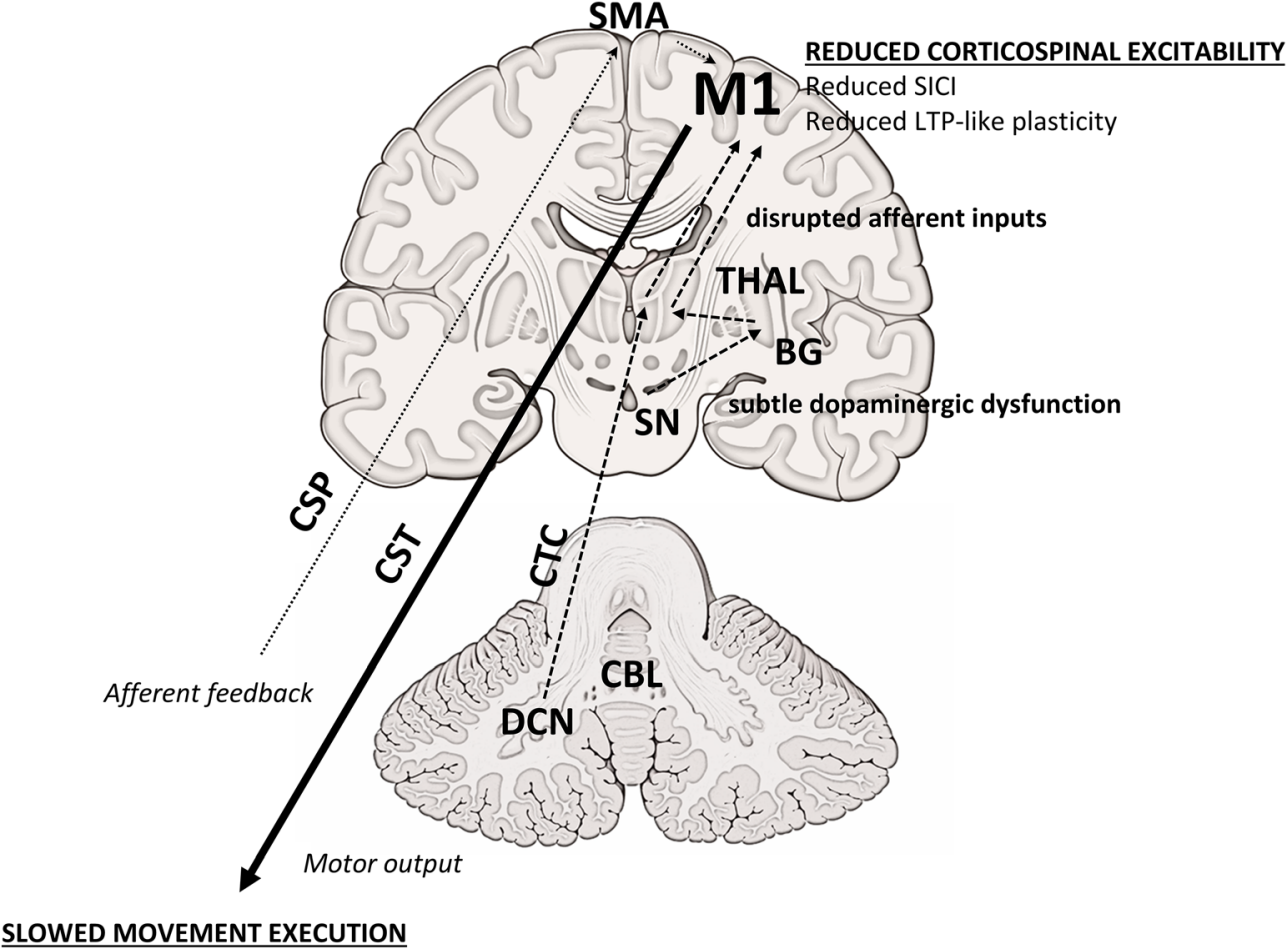
Schematic representation of the putative pathophysiological mechanisms underlying slowed movement execution in essential tremor (ET). The relationship between reduced corticospinal excitability and slowed movement execution (both in bold capital letters and underlined) is emphasized. Other primary motor cortex (M1) alterations, such as reduced short‐interval intracortical inhibition (SICI) and decreased long‐term potentiation (LTP)‐like plasticity (shown in lowercase letters not bold), appear as neurophysiological abnormalities in ET that do not directly relate to movement execution. The diagram highlights that the neurophysiological alterations in M1 may arise from disrupted afferent inputs from subcortical structures (dashed lines), that is, (i) the basal ganglia, possibly due to subtle dopaminergic tone reduction or (ii) altered cerebellar input, via the cerebello‐thalamo‐cortical (CTC) pathway. Note that at the thalamic level, the projections from the basal ganglia and the cerebellum converge to the same areas (here depicted separately for graphic purposes). An additional, though less likely, hypothesis is that the reduced excitability of M1 reflects a compensatory mechanism to mitigate tremor triggered by afferent feedback traveling along the central sensory pathways (CSP), processed in the supplementary motor area (SMA), and relayed back to M1. BG, basal ganglia; CBL, cerebellum; CST, corticospinal tract; DCN, deep cerebellar nuclei; SN, substantia nigra; THAL, thalamus. [Color figure can be viewed at wileyonlinelibrary.com]

Another innovative aspect of this study concerns the kinematic evaluation of voluntary movement in conjunction with TMS. Namely, patients with slower finger‐tapping (ET‐s) showed a more pronounced decrease in M1 excitability than ET‐ns, as evidenced by higher motor thresholds and a flatter I/O curve (Figs 2 and 3). Conversely, we found no relationship with intracortical inhibition, plasticity changes, and measures of motor performance, and no differences between ET subgroups. These findings suggest that reduced corticospinal excitability may represent a mechanism specifically associated with slowed voluntary movement execution in ET. In contrast, abnormalities in GABA‐Aergic neurotransmission and LTP‐like plasticity in M1 appear to be generalized dysfunctions without a direct link to impaired movement execution in ET. The neurophysiological correlates of altered voluntary movements in ET were evident when considering the data in the whole ET sample and were even more evident in the ET‐s subgroup (Fig. 2). The correlation we found in ET may arise from disrupted afferent inputs to M1, from the basal ganglia or the cerebellum.^70^, ^71^ For example, two recent studies demonstrated that subtle bradykinesia in ET patients^15^, ^18^ may depend on striatal dopaminergic dysfunction in the absence of a clear presynaptic dopaminergic deficit.^25^, ^26^ Again, given the pivotal role of the cerebellum in ET pathophysiology, an altered cerebellar input to M1 is another plausible mechanism influencing the observed excitability changes and kinematic abnormalities in patients.^24^, ^72^ This hypothesis is supported by findings from functional MRI (fMRI) studies, which consistently demonstrated disrupted functional connectivity in the network involving the basal ganglia, cerebellum, and motor areas in ET.^24^, ^72^, ^73^, ^74^, ^75^ An alternative hypothesis is that the correlation between reduced corticospinal excitability and slowed movement execution in ET reflects a compensatory mechanism to mitigate tremor. In line with this, it has been demonstrated that other brain regions, such as the supplementary motor area (SMA), may reduce its drive to M1 to limit the propagation of oscillations.^72^ However, we have not found any relationship with both postural and rest tremor and therefore this hypothesis seems less likely (Fig. 4). Finally, to better interpret the potential pathophysiological relationship between excitability changes of M1 and voluntary movement abnormalities in ET we might consider similar observations in PD. Experimental evidence indicates that in PD a steeper I/O curve was associated with reduced movement velocity.^28^ In the PD context, the relationship may be interpreted as a compensatory mechanism, wherein enhanced corticospinal recruitment may serve to offset diminished input from other regions.^28^, ^76^ Similarly to PD, in the ET context movement slowness could arise from reduced input from the basal ganglia, suggesting an involvement of the nigrostriatal pathway. Consistent with this hypothesis, the M1 neurophysiological changes in PD and in ET could arise from a different severity of the dopaminergic deficit in the two conditions. Therefore, when striatal dopaminergic deficits are mild (ET), they determine lower M1 excitability and primarily contribute to reduced movement; however, a more severe dopaminegic dysfunction (PD) can lead to higher corticospinal excitability, possibly reflecting compensatory mechanisms aimed at mitigating motor slowness.^28^, ^76^ Notably, M1 excitability changes in ET seem to be specifically involved in the pathophysiology of altered voluntary movement execution. Indeed, we found no differences in clinical, kinematic, and neurophysiological measures when considering ET patients according to the presence of rest tremor. In addition, the lack of correlation between rest tremor and TMS measures indicates that parkinsonian soft signs in ET (i.e., subtle bradykinesia and rest tremor) may arise from distinct pathophysiological mechanisms.^20^ Again, the lack of correlation between cognitive scores and TMS measures supports the hypothesis that cognitive impairment in ET likely reflects the involvement of broader cortical and subcortical networks, rather than M1 excitability changes.^29^ Importantly, the distribution of other motor soft signs, such as questionable dystonia and impaired tandem gait, did not differ between ET subgroups, whether classified by motor slowness or rest tremor. This further supports the notion that these clinical features do not appear to be directly linked to the mechanisms underlying altered voluntary movement execution in ET.

### 3.3 Confounds and Limitations

Among the potential limitations, we acknowledge the relatively small sample size, as well as the reduced number of HC compared with ET patients. Demographic characteristics, however, were comparable between ET patients and HC, and ET subgroups, thereby excluding the influence of these factors on our findings. The percentage of patients taking medications was comparable between the subgroups, thus limiting their potential influence on the results (Table 1). Each participant underwent evaluation in a single session, minimizing the possible daily variability in neurophysiological parameters. Notably, we specifically examined motor performance by analyzing finger movements which, unlike proximal limb movements, are not significantly influenced by action tremor.^77^, ^78^ Although the ET patients enrolled in this study did not undergo dopamine transporter (DaT) scanning using single‐photon emission computed tomography (SPECT), they were diagnosed according to the latest clinical criteria and those included in the study have been followed up for several years in our outpatient clinic, thus minimizing the risk of misdiagnosis. Finally, we could not determine whether neurophysiological measures correlate with possible neuroimaging abnormalities in ET, as the MRI scans were not acquired using a standardized morphometric protocol. This aspect was beyond the scope of our study; however, it remains a valuable topic for future investigations.^79^

### 3.4 Conclusions

This study provides novel insights into the pathophysiological role of M1 in ET, highlighting the correlation between M1 excitability changes and voluntary movement execution. Our findings support the hypothesis of bradykinesia‐related networks, suggesting that the variable involvement of motor control structures may lead to similar movement impairments across different diseases.^15^, ^80^ Our results should also be interpreted considering the significant heterogeneity of ET, contributing to the ongoing debate on the pathophysiological differences between ET and ET‐plus.^81^, ^82^, ^83^, ^84^, ^85^ Further investigations are warranted to validate our results, possibly combining not only neurophysiology but also other methodologies, such as neuroimaging, to clarify the mechanisms and circuits underlying impaired voluntary movement execution in ET patients and to enhance the pathophysiological understanding of this condition.

## Author Roles

(1) Research project: A. Conception, B. Organization, C. Execution; (2) Statistical analysis: A. Design, B. Execution, C. Review and Critique; (3) Manuscript: A. Writing of the First Draft, B. Review and Critique.

D.B.: 1B, 1C, 2A, 2B, 3A.

L.A.: 1B, 1C, 2C, 3B.

G.P.: 1B, 1C, 3B.

D.Cos.: 1B, 1C, 3B.

A.C.: 1C.

M.P.: 1C.

M.D.R.: 1C.

S.A.: 1C.

D.Col.: 1C.

A.G.: 1B, 2C, 3B.

M.B.: 1A, 1B, 2C, 3B.

## Financial Disclosures of All Authors (for the Preceding 12 Months)

None.

## Supporting information


**Table S1.** Transcranial magnetic stimulation (TMS) studies investigating primary motor cortex (M1) in patients with essential tremor (ET).
**Table S2.** Kinematic measures of each side in essential tremor (ET) patients and the two ET subgroups: ET‐slowness (ET‐s) and ET no‐slowness (ET‐ns).
**Table S3.** Results of the repeated measures analysis of variance (rmANOVA) performed on the neurophysiological measures between the two essential tremor (ET) subgroups: ET‐slowness (ET‐s) and ET no‐slowness (ET‐ns).
**Table S4.** Clinical, kinematic, and transcranial magnetic stimulation (TMS) measures in the essential tremor‐rest tremor (ET‐r) and essential tremor no‐rest tremor (ET‐nr) subgroups.

## Data Availability

The data that support the findings of this study are available on request from the corresponding author. The data are not publicly available due to privacy or ethical restrictions.
