# Supplementary material for: Pathophysiological Role of Primary Motor Cortex in Essential Tremor
Source: Mov Disord. 2025 Apr 17;40(8):1648–60. doi: 10.1002/mds.30197 (PMC12371654; doi:10.1002/mds.30197)
Supplement: Supplementary file 1 — Table S1. Transcranial magnetic stimulation (TMS) studies investigating primary motor cortex (M1) in patients with essential tremor (ET). Table S2. Kinematic measures of each side in essential tremor (ET) patients and the two ET subgroups: ET‐slowness (ET‐s) and ET no‐slowness (ET‐ns). Table S3. Results of the repeated measures analysis of variance (rmANOVA) performed on the neurophysiological measures between the two essential tremor (ET) subgroups: ET‐slowness (ET‐s) and ET no‐slowness (ET‐ns). Table S4. Clinical, kinematic, and transcranial magnetic stimulation (TMS) measures in the essential tremor‐rest tremor (ET‐r) and essential tremor no‐rest tremor (ET‐nr) subgroups. [file MDS-40-1648-s001.docx]

**Supplementary Table 1. TMS studies investigating primary motor cortex (M1) in patients with essential tremor (ET).**

| **References** | **Participants** | **TMS protocol** | **Results** |
| --- | --- | --- | --- |
| Romeo et al. 1998 (9) | 10 ET vs. 8 HC | Single- and paired-pulse TMS: RMT, AMT, cSP, SICI, ICF, LICI | No significant difference between ET and HC |
| Shukla et al. 2003 (10) | 24 ET vs. 24 HC | Single-pulse TMS: cSP | No significant difference between ET and HC |
| Hellriegel et al. 2012 (13) | 10 ET vs. 10 HC | Single-pulse TMS: RMT, AMT  cTBS: 200 triplet bursts of real cTBS and control cTBS | real cTBS reduced corticospinal excitability only in HC but not in ET |
| Chuang et al. 2014 (7) | 13 ET vs. 18 HC | Single- and paired-pulse TMS: AMT, cSP, SICI, ICF  cTBS: 600 triplet bursts | No change in SICI or cSP in ET but reduced SICI and prolonged cSP following cTBS in HC; reduction in MEP amplitude in both ET and HC, but sustained longer in HC, following cTBS |
| Khedr et al. 2019 (8) | 21 ET vs. 20 HC | Single-pulse TMS: RMT, AMT, cSP, I/O curve | RMT and AMT were significantly decreased compared to HC, and patients had a steeper I/O curve; no difference in cSP between ET and HC. |
| Batra et al. 2022 (12) | 10 ET vs. 20 HC | Single- and paired-pulse TMS: RMT, CMCT, cSP, SICI, ICF.  cTBS: 200 triplet bursts | No significant difference in RMT between ET and HC. Reduction of ICF and cSP duration in ET compared to HC. Following cTBS there was a significant increase of the duration of cSP in ET. |
| Žakelj et al. 2024 (11) | 54 ET (before and after treatment with primidone or propranolol) | Single- and paired-pulse TMS: RMT, AMT, SICI, LICI, ICF, SAI, I/O curve | Primidone was associated with decreased corticospinal excitability, prolongation of cSP, increased LICI, increased SAI and decreased SICI. Propranolol was associated with decreased corticospinal excitability and increased SAI. |

Abbreviations: ET, essential tremor; HC, healthy controls; RMT, resting motor threshold; AMT, active motor threshold; cSP, cortical silent period; SICI, short-interval intracortical inhibition; ICF, intracortical facilitation; LICI, long-interval intracortical inhibition; cTBS, continuous theta-burst stimulation; MEP, motor evoked potential; I/O curve, input/output curve of MEPs; CMCT, central motor conduction time; SAI, short-latency afferent inhibition.

**Supplementary Table 2. Kinematic measures of each side in essential tremor patients (ET) and the two ET subgroups: ET-slowness (ET-s) and ET no-slowness (ET-ns).**

|  | **ET (30)** | | | **ET-s (15)** | | | **ET-ns (15)** | | |
| --- | --- | --- | --- | --- | --- | --- | --- | --- | --- |
|  | **Right** | **Left** | **p** | **Right** | **Left** | **p** | **Right** | **Left** | **p** |
| Rest tremor |  |  |  |  |  |  |  |  |  |
| Amplitude | 0.06±0.06 | 0.06±0.06 | 0.78 | 0.07±0.06 | 0.07±0.07 | 0.82 | 0.04±0.04 | 0.04±0.04 | 1.00 |
| Frequency | 6.16±0.90 | 6.04±1.10 | 0.43 | 6.21±1.00 | 6.11±1.23 | 0.62 | 6.05±0.70 | 5.90±0.90 | 0.88 |
| Postural tremor |  |  |  |  |  |  |  |  |  |
| Amplitude | 0.10±0.07 | 0.10±0.08 | 0.94 | 0.11±0.07 | 0.10±0.08 | 0.94 | 0.09±0.07 | 0.09±0.08 | 0.86 |
| Frequency | 6.02±1.30 | 5.90±1.24 | 0.10 | 5.83±1.45 | 5.76±1.30 | 0.44 | 6.22±1.10 | 6.10±1.20 | 0.08 |
| Kinetic tremor |  |  |  |  |  |  |  |  |  |
| CI | 1.08±0.10 | 1.07±0.07 | 0.06 | 1.11±0.14 | 1.07±0.06 | 0.12 | 1.06±0.03 | 1.07±0.08 | 0.25 |
| D/A | 0.53±0.20 | 0.57±0.30 | 0.60 | 0.53±0.25 | 0.58±0.15 | 0.16 | 0.52±0.14 | 0.50±0.14 | 0.28 |
| Finger tapping |  |  |  |  |  |  |  |  |  |
| N° mov | 41.61±11.03 | 40.96±9.82 | 0.48 | 41.14±11.35 | 39.16±10.16 | 0.09 | 42.08±11.07 | 42.88±9.43 | 0.57 |
| Velocity | 996.26±219.76 | 1053.83±233.28 | 0.16 | 825.68±179.73 | 902.99±167.36 | 0.14 | 1166.85±73.33 | 1215.44±180.73 | 0.76 |
| Amplitude | 47.16±10.61 | 43.25±13.56 | 0.09 | 44.34±10.85 | 41.41±10.29 | 0.25 | 49.98±9.92 | 45.22±16.55 | 0.27 |
| CV | 0.11±0.05 | 0.11±0.05 | 0.82 | 0.11±0.04 | 0.12±0.06 | 0.93 | 0.10±0.05 | 0.10±0.05 | 0.72 |
| Velocity slope | -4.20±5.60 | -6.00±4.28 | 0.21 | -3.56±4.75 | -5.58±3.96 | 0.28 | -4.78±6.46 | -6.44±4.71 | 0.58 |
| Amplitude slope | -0.13±0.30 | -0.09±0.22 | 0.54 | -0.09±0.24 | -0.12±0.28 | 0.63 | -0.18±0.35 | -0.06±0.12 | 0.12 |

Values are expressed as mean ± SD. Brackets contain the number of participants in each subgroup in the table header. p-values were calculated using the Wilcoxon signed-rank test. Abbreviations: ET, essential tremor; ET-s, essential tremor-slowness; ET-ns, essential tremor no-slowness; CI, curvature index; D/A, deceleration/acceleration ratio; N° mov, number of movements; CV, coefficient of variation

**Supplementary Table 3. Results of the rmANOVA performed on the neurophysiological measures between the two ET subgroups: ET-slowness (ET-s) and ET no-slowness (ET-ns).**

|  | **df** | **F** | **p** |
| --- | --- | --- | --- |
| I/O SLOPE |  |  |  |
| GROUP | 1,28 | 2.21 | 0.15 |
| INTENSITY | 4,112 | 58.50 | **<0.01** |
| GROUP × INTENSITY | 4,112 | 4.44 | **<0.01** |
| SICI |  |  |  |
| GROUP | 1,28 | 1.08 | 0.31 |
| ISI | 1,28 | 5.59 | 0.03 |
| GROUP × ISI | 1,28 | 0.45 | 0.51 |
| SAI |  |  |  |
| GROUP | 1,28 | 0.06 | 0.81 |
| ISI | 1,28 | 2.61 | 0.12 |
| GROUP × ISI | 1,28 | 0.25 | 0.62 |
| ITBS |  |  |  |
| GROUP | 1,27 | 0.001 | 0.98 |
| TIME | 2,54 | 1.09 | 0.34 |
| GROUP × TIME | 2,54 | 2.99 | 0.06 |

Only values that are significant after FDR correction are highlighted in bold. Abbreviations: I/O slope, input/output slope of motor evoked potentials; SICI, short-interval intracortical inhibition; ISI, interstimulus interval; SAI, short-latency afferent inhibition; iTBS, intermittent theta-burst stimulation.

**Supplementary Table 4. Clinical, kinematic and TMS measures in essential tremor-rest tremor (ET-r) and in essential tremor no-rest tremor (ET-nr) subgroups.**

|  | **ET-r (13)** | **ET-nr (17)** | **p** |
| --- | --- | --- | --- |
| Age (y) | 68.38±8.81 | 65.18±11.82 | 0.74 |
| Sex | 8M (61.5%) | 12M (70.5%) | 0.71 |
| Tremor duration (y) | 15.31±12.66 | 19.65±12.80 | 0.30 |
| FTM-TRS | 31.23±17.27 | 20.00±9.35 | 0.07 |
| MDS-UPDRS-III | 11.77±7.05 | 7.88±6.63 | 0.13 |
| MoCA | 23.38±2.63 | 25.24±2.88 | 0.06 |
| FAB | 16.23±2.24 | 16.94±1.25 | 0.50 |
| Soft signs |  |  |  |
| B | 8 (61.5%) | 7 (41.2%) | 0.46 |
| MCI | 7 (53.8%) | 4 (23.5%) | 0.13 |
| QD | 6 (46.2%) | 6 (35.3%) | 0.71 |
| ITG | 3 (23.0%) | 2 (11.8%) | 0.63 |
| Therapy |  |  |  |
| Propranolol | 7 (53.8%) | 7 (41.2%) | 0.71 |
| Topiramate | 1 (7.7%) | 2 (11.8%) | 1.00 |
| BDZ | 4 (30.8%) | 1 (5.9%) | 0.63 |
| Gabapentin | 3 (23.1%) | 2 (11.8%) | 0.14 |
| No therapy | 2 (15.4%) | 7 (41.2%) | 0.23 |
| RMT | 51.90±8.00 | 50.82±8.89 | 0.95 |
| AMT | 43.08±6.92 | 40.59±7.74 | 0.24 |
| I/O slope | 3.24±2.48 | 3.24±2.50 | 0.46 |
| SICI |  |  |  |
| 2 ms | 0.60±0.39 | 0.70±0.28 | 1.00 |
| 4 ms | 0.75±0.38 | 0.83±0.36 | 1.00 |
| SAI |  |  |  |
| 22 ms | 0.45±0.20 | 0.55±0.23 | 1.00 |
| 24 ms | 0.49±0.24 | 0.62±0.27 | 1.00 |
| Post iTBS |  |  |  |
| 5 min | 0.90±0.39 | 1.01±0.30 | 1.00 |
| 15 min | 0.91±0.35 | 1.11±0.48 | 1.00 |
| 30 min | 1.12±0.55 | 1.06±0.43 | 1.00 |
| Postural tremor |  |  |  |
| Amplitude | 0.12±0.07 | 0.08±0.07 | 0.04 |
| Frequency | 5.97±1.19 | 5.96±1.31 | 0.93 |
| Kinetic tremor |  |  |  |
| CI | 1.08±0.06 | 1.08±0.08 | 0.66 |
| D/A | 0.52±0.15 | 0.54±0.18 | 0.76 |
| Finger tapping |  |  |  |
| N° mov | 40.49±10.05 | 42.47±11.95 | 0.98 |
| Velocity | 936.72±261.80 | 1041.80±176.19 | 0.22 |
| Amplitude | 44.09±11.58 | 49.51±9.48 | 0.25 |
| CV | 0.11±0.06 | 0.10±0.03 | 0.72 |
| Velocity slope | -4.39±4.45 | -4.01±6.48 | 1.00 |
| Amplitude slope | -0.09±0.24 | -0.17±0.34 | 0.71 |

Values are expressed as mean ± SD. The number of participants in each subgroup are in parentheses in the column headers, and the percentages are stated within the table rows. For each soft sign and drug, the number of patients is specified. Short-interval intracortical inhibition (SICI), short-latency afferent inhibition (SAI) and post-intermittent theta-burst stimulation (iTBS) values are expressed as the ratio of conditioned to unconditioned motor evoked potentials amplitudes. Tremor values are expressed as the mean of both sides, and finger-tapping values refer to the right (dominant) hand. p-values for SICI, SAI, and synaptic plasticity were obtained from post-hoc tests performed with Bonferroni correction, while the other p-values were calculated using the Mann-Whitney U test or the Fisher's exact test where appropriate. Only values that are significant after false discovery rate correction are highlighted in bold. Abbreviations: ET-r, essential tremor–rest tremor; ET-nr, essential tremor no-rest tremor; M, male; FTM-TRS, Fahn-Tolosa-Marin Tremor Rating Scale; MDS-UPDRS-III, Movement Disorder Society-sponsored revision of the Unified Parkinson’s Disease Rating Scale (part III); MoCA, Montreal Cognitive Assessment; FAB, Frontal Assessment Battery; B, bradykinesia; MCI, mild cognitive impairment; QD, questionable dystonia; ITG, impaired tandem gait, BDZ, benzodiazepines; RMT, resting motor threshold; AMT, active motor threshold; I/O slope, input/output slope of motor evoked potentials; SICI, short-interval intracortical inhibition; SAI, short-latency afferent inhibition; iTBS, intermittent theta-burst stimulation; CI, curvature index; D/A, deceleration/acceleration ratio; N° mov, number of movements; CV, coefficient of variation.
